# Sex differences in acute kidney injury requiring dialysis

**DOI:** 10.1186/s12882-018-0937-y

**Published:** 2018-06-08

**Authors:** Joel Neugarten, Ladan Golestaneh, Nitin V. Kolhe

**Affiliations:** 1 Department of Medicine, Nephrology Division, Montefiore Medical Center, Albert Einstein College of Medicine, 111 E. 210 St, Bronx, NY 10467 USA; 2 Department of Renal Medicine, Royal Derby Hospital, Uttoxeter Road, Derby, DE22 3NE UK

**Keywords:** Gender, Sex, Acute kidney injury, Acute renal failure, AKI

## Abstract

**Background:**

Female sex has been included as a risk factor in models developed to predict the risk of acute kidney injury (AKI) associated with cardiac surgery, aminoglycoside nephrotoxicity and contrast-induced nephropathy. The commentary acompanying the Kidney Disease Improving Global Outcomes Clinical Practice Guideline for Acute Kidney Injury concludes that female sex is a shared susceptibility factor for acute kidney injury based on observations that female sex is associated with the development of hospital-acquired acute kidney injury. In contrast, female sex is reno-protective in animal models. In this context, we sought to examine the role of sex in hospital-associated acute kidney injury in greater detail.

**Methods:**

We utilized the Hospital Episode Statistics database to calculate the sex-stratified incidence of AKI requiring renal replacement therapy (AKI-D) among 194,157,726 hospital discharges reported for the years 1998–2013. In addition, we conducted a systematic review of the English literature to evaluate dialysis practices among men versus women with AKI.

**Results:**

Hospitalized men were more likely to develop AKI-D than hospitalized women (OR 2.19 (2.15, 2.22) *p* < 0.0001). We found no evidence in the published literature that dialysis practices differ between men and women with AKI.

**Conclusions:**

Based on a population of hospitalized patients which is more than 3 times larger than all previously published cohorts reporting sex-stratified AKI data combined, we conclude that male sex is associated with an increased incidence of hospital-associated AKI-D. Our study is among the first reports to highlight the protective role of female gender in AKI.

## Background

Sexual dimorphism is a well-recognized feature of chronic progressive kidney disease. [1–5] Female sex is thought to play a protective role, meditated by differences in the hormonal milieu. [1–5] Although less well appreciated, sexual dimorphism has also been established in the development of ischemic acute kidney injury (AKI). [6] Animal models have consistently shown a protective effect of female sex on the development of AKI after ischemia-reperfusion injury. [7–19] Contrary to these experimental observations, the direction of sexual dimorphism has been reported to be reversed in AKI in humans. [20] Female sex has been identified as a risk factor in predictive models of acute kidney injury associated with cardiac surgery, aminoglycoside or radio-contrast administration, and rhabdomyolysis. [21–24]. In addition, the commentary accompanying the Kidney Disease Improving Global Outcomes (KDIGO) Clinical Practice Guideline for Acute Kidney Injury (online appendix A-F) states that female sex is among the shared susceptibility factors that confer a higher risk of AKI. [20] This conclusion is based on studies demonstrating that women are more likely than men to develop hospital-acquired AKI. [20] The guideline endorses the findings that female sex is associated with a higher risk for AKI in studies of cardiac surgery-associated AKI, contrast-induced nephropathy, and aminoglycoside nephropathy. [20] On the basis of these observations, the commentary concludes that, “contrary to most chronic kidney disease disorders, it is the female gender that carries a higher risk for AKI.” [20] However, the guideline goes on to qualify this conclusion by recognizing that males predominate in reports of AKI complicating infections with HIV, malaria, leptospirosis and other community acquired forms of AKI.

We have previously challenged the generally held consensus that female sex is an independent risk factor for cardiac surgery-associated AKI and for aminoglycoside nephrotoxicity. [6, 25] In the present study, we sought to explore the relationship between sex and hospital-associated AKI requiring renal replacement therapy in greater detail by examining this relationship in a large administrative database of over 194 million hospital encounters which is more than 3 times larger than all of the published cohorts reporting sex-stratified AKI incidence data combined.

## Methods

### Data source

The data source and methodology have been described in detail in earlier publications. [26, 27] In brief, we utilized the Health & Social Care Information Centre Hospital Episodes Statistics database to compile information about all discharges from United Kingdom National Health Service hospitals reported for the fiscal years 1998–2013. Episodes of AKI were identified among finished discharge spells, defined as the total continuous completed stay of a patient using a hospital bed in premises controlled by a health care provider, during which medical care is the responsibility of one or more consultants. Data were used to calculate the sex-stratified incidence of acute kidney injury requiring renal replacement therapy (AKI-D) and in-hospital fatality rates during the study period. To account for any bias introduced by patients with unknown or missing sex classification, these patients were alternately deemed to be all male or all female for the purpose of calculating sex-stratified incidence of AKI-D and mortality rates.

### Identification of AKI

The procedures used to identify patients with AKI-D have been described in detail in earlier publications. [26, 27] In brief, up to 20 Office of Population and Census Surveys Classification of Interventions and Procedures version 4 codes were analyzed to identify cases of AKI-D, coded × 40.2 (hemodialysis) or × 40.4 (hemofiltration). Patients with preexisting end stage renal disease or stage 5 chronic kidney disease were excluded on the basis of codes N18.5 (stage 5 chronic kidney disease), N18.6 (end stage renal disease), L74.2 (arteriovenous fistula) or L74.3 (arteriovenous graft). This definition included patients who developed AKI within the first 48 h of admission to the hospital (community-acquired AKI) and patients who developed AKI later in their hospital course (hospital-acquired). Data for the period April, 1998 until March, 2013 were divided into 3 five year periods for analysis (April 1988 to March 2003, April 2003 to March 2008 and April 2008 to March 2013.

### Statistical analysis

Statistical analysis was performed utilizing the chi-square test, odds ratio chi square test and binary logistic regression analysis.

### Literature search strategy and selection criteria

**Fig. 1:**
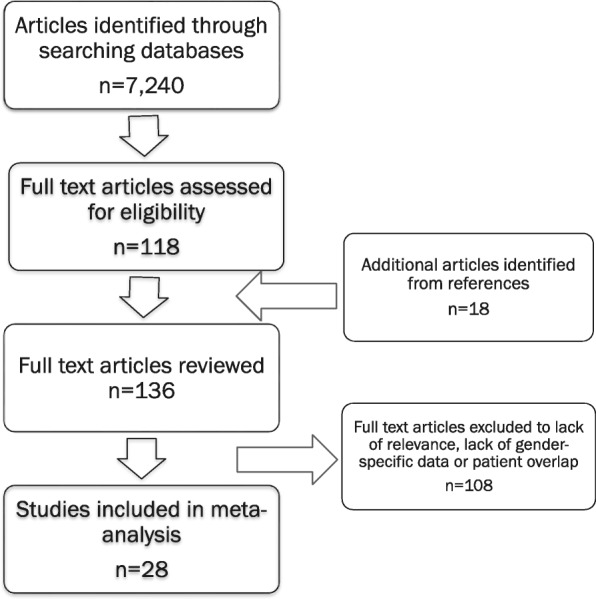
Search strategy

We conducted a systematic review of the English literature to evaluate dialysis practices among hospitalized men compared to hospitalized women with acute kidney injury. We search PubMed and EMBASE for English-language articles published between January 1, 1988 and December 31, 2016. The following medical subject heading terms were used: (male, female, gender, or sex), (acute kidney injury, or acute renal failure) and dialysis. Titles and abstracts of the articles found in the search were reviewed to identify eligible studies. Full text versions of selected studies were analyzed in detail. We also examined the bibliographies of recovered articles for additional references. Any case control or cohort study of hospitalized patients with AKI in which dialysis practices were reported according to sex were eligible for inclusion. All studies were examined for duplication of data. Attention was given to reporting clinical center, the years covered and overlap with larger regional or national databases (Fig. 1).

## Results

### Incidence of AKI-D among hospitalized men and women

The incidence of AKI requiring renal replacement therapy (AKI-D) among 194,157,726 patients with finished discharge spells reported in the Hospital Episodes Statistics database for the years 1998–2013 was 0.334/1000 hospital discharges (0.494/1000 discharges in men and 0.226/1000 discharges in women). Men accounted for 43.4% of all hospital discharges and 61.7% of the AKI-D cohort. Maternity admissions were responsible for 0.1% of the AKI-D cohort. Additional demographic details for the study population have previously been reported. [26]

Males were 2.19 times more likely to develop AKI-D than females (OR 2.19 (2.15, 2.22), *p* < 0.0001). The data were not significantly altered when all patients with missing sex classification were alternately deemed to be all male or all female. The relative risk of AKI-D in males compared to females remained constant over the 15 year study period (2.10 in 1998–2003 versus 2.05 in 2008–2013).

### Literature review of Dialysis practices in men and women with AKI

We identified 9 studies in which the decision to initiate renal replacement therapy (RRT) rather than pursue conservative, non-dialytic management was assessed in men versus women with AKI [28–36]. The severity of AKI in the two comparison cohorts was judged to be comparable based on AKIN criteria (3 studies), RIFLE criteria (3 studies), KDIGO criteria (1 study), serum creatinine level (1 study), or investigator-defined indications for RRT (1 study). Two of these studies utilized propensity scoring and multivariate analysis. The country of origin of these studies was Asia (2), Europe (3), the United States (2), Australia, and multinational. In 4 studies, women were more likely than men to receive RRT rather than conservative, non-dialytic management of AKI despite comparable severity of AKI. In 4 other studies no difference in dialysis practices were observed between the sexes. In only one study were men more likely than women to undergo RRT rather than conservative non-dialytic management of AKI despite comparable severity of AKI.

We identified 19 studies which assessed early versus late initiation of RRT in men versus women with AKI. [34, 37–54] Early versus late initiation of RRT was defined based on the following criteria: BUN level (6 studies), the time from a specified event such as onset of oliguria or satisfying indications for RRT (8 studies), AKIN criteria (1 study), RIFLE criteria (5 studies), or serum creatinine level (2 studies); in several cases more than one criterion was utilized in the same study. The country of origin of these studies was Europe (6), Asia (7), the Americas (5) and multinational. Five studies found that women initiated dialysis earlier than men despite comparable severity of AKI. Thirteen studies found no difference in the timing of RRT between men and women with comparable severity of AKI. The remaining study found that dialysis was initiated earlier in women than in men when serum creatinine level was used to define the timing of dialytic intervention, but found no difference between the sexes when other criteria were used. We were unable to identify any study that found than men with AKI underwent dialysis earlier than women with comparable severity of AKI.

## Discussion

Sexual dimorphism is a well-recognized feature of chronic progressive kidney disease disease. [1–5] Although not well appreciated, sexual dimorphism has also been clearly established in ischemic AKI. [6] In contrast to CKD, where female sex is reno-protective, the direction of sexual dimorphism has been reported to be reversed in several specific forms of acute kidney injury in humans, including contrast-associated nephropathy, aminoglycoside nephrotoxicity and cardiac surgery-associated AKI.[20] On the basis of these observations, the KDIGO AKI guideline concludes that female sex is a risk factor for AKI. [20] This conclusion, however, was qualified by the observation that males predominate in reports of AKI complicating infections with HIV, malaria, leptospirosis and other community acquired forms of AKI.

The conclusion that female sex is an independent risk factor for cardiac surgery-associated AKI is contradicted by our previously published findings. [6] The conclusion that female sex is associated with an increased risk of aminoglycoside nephrotoxicity is primarily based on a small number of subjects studied by one group of investigators and contradicted by the findings of other investigators, including ourselves. [25] Although the incidence of contrast-induced nephropathy has frequently been found to be greater in women than in men [55, 56], it has been suggested that this association may merely reflect differences in administered contrast volume when adjusted for body surface area. [57]

In this study, we report the sex-stratified incidence of AKI-D among hospitalized patients in a large national database which is more than 3 times larger than all of the published cohorts reporting sex-stratified AKI incidence data combined. We found that males were 2.2 times more likely to develop AKI-D than females. Our data are similar to that previously reported from the United States. Hsu et al. [58] utilized the National Inpatient Sample to identify patients with dialysis-requiring AKI among 24 million hospitalizations, constituting a nationally representative sample of all hospitalizations in the United States between the years 2007 and 2009. Men were 1.9 times more likely to develop AKI-D than women. Similarly, Waiker et al. [59] utilized the same database to identify AKI-D among nearly 16 million hospitalizations between 2000 and 2003. Men were 1.9 times more likely to develop AKI-D than women. However, the National Inpatient Sample does not include patients hospitalized in Veterans Administration facilities. In this regard, Cronin et al. [60] studied a cohort of over 1.6 million predominantly male patients hospitalized in Veterans Administration facilities between 2003 and 2012 and identified those with AKI by KDIGO criteria and by dialysis procedure codes. By multivariate analysis, male sex was associated with a 1.27 higher risk of stage 1–3 AKI and a 2 fold greater risk of AKI-D. Although data on the relative incidence of AKI-D in men versus women have previously been reported from large administrative databases, the implications of these data have not been widely recognized. Under-recognition of the protective role of female gender in the development of AKI is reflected in the KDIGO Guideline on AKI. Our study is among the first reports to highlight the protective role of female gender in AKI in humans.

There is strong experimental basis to support our hypothesis that female sex is reno-protective in AKI. [5, 7–19] We suggest that this reno-protection is mediated by effects of sex hormones on cellular processes instrumental in the pathogenesis of AKI, analogous to our suggestion that sex hormones mediate the beneficial effects of female sex on the course of chronic kidney disease. [1–5] In experimental models of ischemic AKI, females show less severe functional renal impairment and less histologic damage after ischemia-reperfusion injury. [7–19] Oophorectomy or testosterone administration has been shown to exert deleterious effects whereas castration or exogenous estrogen has been shown to be reno-protective. [9, 19] Numerous hypotheses have been proposed to explain these observations. [5, 9, 19] Sex-related differences in the generation of nitric oxide, in the synthesis and vascular response to endothelin-1, and in the renal hemodynamic response to angiotensin II have been demonstrated in experimental models and in human subjects. [9, 19] In addition, differences between the sexes in the inflammatory, hemodynamic and humoral response to ischemia-reperfusion injury have been observed. [5, 7, 9–19]

We also found that non-dialysis requiring AKI was 1.53 times more likely to develop in men than in women (OR 1.53 (1.52,1.54), *p* < 0. 0001). However, we did not report this finding in light of concerns about the validity of using administrative codes to identify non-dialysis requiring AKI in men versus women. In this regard, Waiker et al. [61] reported that the sensitivity of administrative codes to identify a 100% change in serum creatinine level during hospital admission was significantly greater in men than in women. In contrast, numerous studies have established the high sensitivity, specificity, positive predictive value and negative predictive value of diagnostic codes to identify AKI-D in a variety of administrative databases [61–65]. These indices generally exceeded 90%. [62] Not only do diagnostic codes used to identify AKI-D have a greater accuracy than those used to identify AKI, they are also unlikely to be subject to miscoding based on the sex of the patient. Yet the fact remains that, despite the objective basis for dialysis coding, the actual decision to initiate dialysis by the clinician is essentially a subjective one.

Taking into account this inherent subjectivity, we must first consider 2 inter-related issues before we can conclude that the relative incidence of AKI-D in men versus women reflects a genuine difference in the incidence of severe AKI. First, whether or not there are sex-related influences on the decision to forgo RRT in favor of conservative non-dialytic therapy and second, whether or not there are sex-related differences in the timing of RRT despite equivalent severity of AKI and equivalent indications for dialytic intervention. Later initiation of dialysis in women than in men would introduce the competing risk of mortality from underlying illnesses and would reduce the number of women with AKI at risk for dialysis. Both of these aforementioned factors might increase the incidence of AKI-D in men versus women in the absence of a genuine difference in the incidence of severe AKI. To address these issues, we performed a systematic review of differences between the sexes in dialysis practices in patients with AKI.

We identified 9 studies in which the decision to initiate RRT rather than pursue conservative non-dialytic management was assessed in men versus women with identical severity of AKI [28–36]. These studies do not address the issue of whether or not the incidence of AKI-D is influenced by sex, but instead address the question of whether or not dialysis practices differ between men and women matched for identical severity of AKI. After propensity score matching of 545 patients with AKI who underwent RRT with an equal number of patients with AKI who did not undergo RRT, Wilson et al. [36] reported that dialysis was nearly twice as likely to be initiated in women compared to men. Similarly, 3 other studies found that women with AKI were more likely to receive RRT than men. In contrast, Vaara et al. [34] created a propensity score model for preemptive RRT (*n* = 105) versus no RRT (*n* = 2540) and found no difference between the sexes in the provision of RRT to patients with AKI in the absence of classical indications for dialysis. Similarly, 3 other studies found no difference between the sexes in the provision of RRT in AKI. In only one study were men more likely to undergo RRT compared to women. [28]

We identified 19 studies which assessed early versus late initiation of RRT in men versus women matched for severity of AKI. [34, 37–54]. Again, these studies do not address the issue of whether or not the incidence of AKI-D is influenced by sex, but instead address the question of whether or not dialysis practices differ between men and women matched for identical severity of AKI. Chou et al. [41] utilized propensity scoring and logistic regression analysis in 370 patients with sepsis and AKI treated in surgical intensive care units and found that women, as compared to men, were 50% more likely to undergo early initiation of dialysis as assessed by RIFLE criteria. Four additional studies found that women initiated dialysis earlier than men. In contrast, Liu et al. [49] concluded that sex was not an independent predictor of early versus late dialysis using multivariate analysis despite the finding that male sex was associated with early initiation of RRT on univariate analysis. Twelve additional studies found no difference in the timing of RRT initiation between men and women. The remaining study by Bagshaw et al. [37] found that dialysis was initiated earlier in women with AKI than in men with AKI when serum creatinine level was used to define timing of dialytic intervention but found no difference between the sexes when other criteria were utilized. We were unable to identify any study that found than men with AKI underwent dialysis earlier than women. Thus, available data provide no evidence that men with AKI are more likely to undergo RRT or more likely to initiate RRT earlier than women with comparable severity of AKI. These data support our conclusion that the greater incidence of AKI-D in men compared to women truly reflects a difference in the incidence of severe AKI between the sexes.

Thus, our data clearly establishes an increased incidence of AKI-D in hospitalized men compared to women. Although we suggest that this difference reflects an increased incidence of severe AKI in men, alternative explanations may exist. Since dialysis is not a hard outcome, our conclusion may reflect differences in dialysis practices between the sexes; however, our systematic review did not support this contention. But the fact remains that none of the analyzed studies were specifically designed to compare dialysis practices in men versus women. Furthermore, dialysis may be initiated for reasons other than AKI severity, including refractory volume overload and intoxications. Local practices may differ and introduce bias, and dialysis practices have changed over time, although we consistently found a greater incidence of AKI-D in men in each time interval studied. Since AKI is not a disease but a heterogeneous group of disorders characterized by a reduction in renal function, all etiologies of AKI may not exhibit similar sexual dimorphism. Lastly, we cannot determine whether sexual dimorphism in hospital-associated AKI is driven by community-acquired AKI or hospital-acquired AKI, or both.

## Conclusions

In a large national database which is more than 3 times larger than all of the published cohorts reporting sex-stratified AKI incidence data combined, men were 2.19 times more likely to develop AKI-D than women. This finding questions the generally held belief, expressed in the KDIGO Guideline for AKI, that female sex is a significant risk factor for AKI. On the contrary, and consistent with observations in animal models, male sex is associated with an increased incidence of hospital-associated AKI-D. Our study is among the first reports to highlight the protective role of female sex in AKI in humans.

## Abbreviations

AKI: Acute Kidney injury
AKI-D: Acute kidney injury requiring dialysis
KDIGO: Kidney Disease Improving Global Outcomes
OR: Odds ratio
RRT: Renal replacement therapy

## References

[1] Neugarten J (2002). Gender and the progression of renal disease. J Am Soc Nephrol.

[2] Neugarten J, Acharya A, Silbiger SR (2000). Effect of gender on the progression of nondiabetic renal disease: a meta-analysis. J Am Soc Nephrol.

[3] Neugarten J, Golestaneh L (2013). Gender and the prevalence and progression of renal disease. Adv Chronic Kidney Dis.

[4] Neugarten J, Silbiger SR (1995). Effects of sex hormones on mesangial cells. Am J Kidney Dis.

[5] Dubey RK, Jackson EK (2001). Estrogen-induced cardiorenal protection: potential cellular, biochemical, and molecular mechanisms. Am J Physiol Renal Physiol.

[6] Neugarten J (2012). Renal BOLD-MRI and assessment for renal hypoxia. Kidney Int.

[7] Fekete A, Vannay A, Ver A, Rusai K, Muller V, Reusz G, Tulassay T, Szabo AJ (2006). Sex differences in heat shock protein 72 expression and localization in rats following renal ischemia-reperfusion injury. Am J Physiol Renal Physiol.

[8] Fekete A, Vannay A, Ver A, Vasarhelyi B, Muller V, Ouyang N, Reusz G, Tulassay T, Szabo AJ (2004). Sex differences in the alterations of Na(+), K(+)-ATPase following ischaemia-reperfusion injury in the rat kidney. J Physiol.

[9] Hutchens MP, Dunlap J, Hurn PD, Jarnberg PO (2008). Renal ischemia: does sex matter?. Anesth Analg.

[10] Hutchens MP, Fujiyoshi T, Komers R, Herson PS, Anderson S (2012). Estrogen protects renal endothelial barrier function from ischemia-reperfusion in vitro and in vivo. Am J Physiol Renal Physiol.

[11] Kang KP, Lee JE, Lee AS, Jung YJ, Kim D, Lee S, Hwang HP, Kim W, Park SK (2014). Effect of gender differences on the regulation of renal ischemia-reperfusion-induced inflammation in mice. Mol Med Rep.

[12] Kher A, Meldrum KK, Wang M, Tsai BM, Pitcher JM, Meldrum DR (2005). Cellular and molecular mechanisms of sex differences in renal ischemia-reperfusion injury. Cardiovasc Res.

[13] Muller V, Losonczy G, Heemann U, Vannay A, Fekete A, Reusz G, Tulassay T, Szabo AJ (2002). Sexual dimorphism in renal ischemia-reperfusion injury in rats: possible role of endothelin. Kidney Int.

[14] Park KM, Kim JI, Ahn Y, Bonventre AJ, Bonventre JV (2004). Testosterone is responsible for enhanced susceptibility of males to ischemic renal injury. J Biol Chem.

[15] Rodriguez F, Nieto-Ceron S, Fenoy FJ, Lopez B, Hernandez I, Martinez RR, Soriano MJ, Salom MG (2010). Sex differences in nitrosative stress during renal ischemia. Am J Physiol Regul Integr Comp Physiol.

[16] Satake A, Takaoka M, Nishikawa M, Yuba M, Shibata Y, Okumura K, Kitano K, Tsutsui H, Fujii K, Kobuchi S (2008). Protective effect of 17beta-estradiol on ischemic acute renal failure through the PI3K/Akt/eNOS pathway. Kidney Int.

[17] Takayama J, Takaoka M, Sugino Y, Yamamoto Y, Ohkita M, Matsumura Y (2007). Sex difference in ischemic acute renal failure in rats: approach by proteomic analysis. Biol Pharm Bull.

[18] Tanaka R, Tsutsui H, Kobuchi S, Sugiura T, Yamagata M, Ohkita M, Takaoka M, Yukimura T, Matsumura Y (2012). Protective effect of 17beta-estradiol on ischemic acute kidney injury through the renal sympathetic nervous system. Eur J Pharmacol.

[19] Metcalfe PD, Meldrum KK (2006). Sex differences and the role of sex steroids in renal injury. J Urol.

[20] KDIGO Clinical Practice Guideline for Acute Kidney Injury. Kidney Int 2012, 2(Supplement 1):1–138; Online Appendices A-F.

[21] Mehran R, Aymong ED, Nikolsky E, Lasic Z, Iakovou I, Fahy M, Mintz GS, Lansky AJ, Moses JW, Stone GW (2004). A simple risk score for prediction of contrast-induced nephropathy after percutaneous coronary intervention: development and initial validation. J Am Coll Cardiol.

[22] Moore RD, Smith CR, Lipsky JJ, Mellits ED, Lietman PS (1984). Risk factors for nephrotoxicity in patients treated with aminoglycosides. Ann Intern Med.

[23] Neugarten J, Sandilya S, Singh B, Golestaneh L (2016). Sex and the risk of AKI following cardio-thoracic surgery: a meta-analysis. Clin J Am Soc Nephrol.

[24] McMahon GM, Zeng X, Waikar SS (2013). A risk prediction score for kidney failure or mortality in rhabdomyolysis. JAMA Intern Med.

[25] Neugarten J, Golestaneh L (2016). The effect of gender on aminoglycoside-associated nephrotoxicity. Clin Nephrol.

[26] Kolhe NV, Muirhead AW, Wilkes SR, Fluck RJ, Taal MW (2015). National trends in acute kidney injury requiring dialysis in England between 1998 and 2013. Kidney Int.

[27] Kolhe NV, Muirhead AW, Wilkes SR, Fluck RJ, Taal MW (2016). The epidemiology of hospitalised acute kidney injury not requiring dialysis in England from 1998 to 2013: retrospective analysis of hospital episode statistics. Int J Clin Pract.

[28] Clec'h C, Darmon M, Lautrette A, Chemouni F, Azoulay E, Schwebel C, Dumenil AS, Garrouste-Orgeas M, Goldgran-Toledano D, Cohen Y (2012). Efficacy of renal replacement therapy in critically ill patients: a propensity analysis. Crit Care.

[29] Gaudry S, Ricard JD, Leclaire C, Rafat C, Messika J, Bedet A, Regard L, Hajage D, Dreyfuss D (2014). Acute kidney injury in critical care: experience of a conservative strategy. J Crit Care.

[30] O'Leary JG, Wong F, Reddy KR, Garcia-Tsao G, Kamath PS, Biggins SW, Fallon MB, Subramanian RM, Maliakkal B, Thacker L (2017). Gender-specific differences in baseline, peak, and Delta serum creatinine: the NACSELD experience. Dig Dis Sci.

[31] Schneider AG, Eastwood GM, Seevanayagam S, Matalanis G, Bellomo R (2012). A risk, injury, failure, loss, and end-stage renal failure score-based trigger for renal replacement therapy and survival after cardiac surgery. J Crit Care.

[32] Schneider AG, Uchino S, Bellomo R (2012). Severe acute kidney injury not treated with renal replacement therapy: characteristics and outcome. Nephrol Dial Transplant.

[33] Tian H, Sun T, Hao D, Wang T, Li Z, Han S, Qi Z, Dong Z, Lv C, Wang X. The optimal timing of continuous renal replacement therapy for patients with sepsis-induced acute kidney injury. Int Urol Nephrol. 2014;46(10):2009–14. 10.1007/s11255-014-0747-5 24913907

[34] Vaara ST, Reinikainen M, Wald R, Bagshaw SM, Pettila V, Group FS (2014). Timing of RRT based on the presence of conventional indications. Clin J Am Soc Nephrol.

[35] Wang F, Hong D, Wang Y, Feng Y, Wang L, Yang L, Consortium IAbC (2017). Renal replacement therapy in acute kidney injury from a Chinese cross-sectional study: patient, clinical, socioeconomic and health service predictors of treatment. BMC Nephrol.

[36] Wilson FP, Yang W, Machado CA, Mariani LH, Borovskiy Y, Berns JS, Feldman HI (2014). Dialysis versus nondialysis in patients with AKI: a propensity-matched cohort study. Clin J Am Soc Nephrol.

[37] Bagshaw SM, Uchino S, Bellomo R, Morimatsu H, Morgera S, Schetz M, Tan I, Bouman C, Macedo E, Gibney N (2009). Timing of renal replacement therapy and clinical outcomes in critically ill patients with severe acute kidney injury. J Crit Care.

[38] Boussekey N, Capron B, Delannoy PY, Devos P, Alfandari S, Chiche A, Meybeck A, Georges H, Leroy O (2012). Survival in critically ill patients with acute kidney injury treated with early hemodiafiltration. Int J Artif Organs.

[39] Carl DE, Grossman C, Behnke M, Sessler CN, Gehr TW (2010). Effect of timing of dialysis on mortality in critically ill, septic patients with acute renal failure. Hemodial Int.

[40] Chon GR, Chang JW, Huh JW, Lim CM, Koh Y, Park SK, Park JS, Hong SB (2012). A comparison of the time from sepsis to inception of continuous renal replacement therapy versus RIFLE criteria in patients with septic acute kidney injury. Shock.

[41] Chou YH, Huang TM, Wu VC, Wang CY, Shiao CC, Lai CF, Tsai HB, Chao CT, Young GH, Wang WJ (2011). Impact of timing of renal replacement therapy initiation on outcome of septic acute kidney injury. Crit Care.

[42] De Corte W, Vanholder R, Dhondt AW, De Waele JJ, Decruyenaere J, Danneels C, Claus S, Hoste EA (2011). Serum urea concentration is probably not related to outcome in ICU patients with AKI and renal replacement therapy. Nephrol Dial Transplant.

[43] Elahi MM, Lim MY, Joseph RN, Dhannapuneni RR, Spyt TJ (2004). Early hemofiltration improves survival in post-cardiotomy patients with acute renal failure. Eur J Cardiothorac Surg.

[44] Garcia-Fernandez N, Perez-Valdivieso JR, Bes-Rastrollo M, Vives M, Lavilla J, Herreros J, Monedero P, Gedrcc (2011). Timing of renal replacement therapy after cardiac surgery: a retrospective multicenter Spanish cohort study. Blood Purif.

[45] Gettings LG, Reynolds HN, Scalea T (1999). Outcome in post-traumatic acute renal failure when continuous renal replacement therapy is applied early vs. late. Intensive Care Med.

[46] Iyem H, Tavli M, Akcicek F, Buket S (2009). Importance of early dialysis for acute renal failure after an open-heart surgery. Hemodial Int.

[47] Ji Q, Mei Y, Wang X, Feng J, Cai J, Zhou Y, Sun Y, Xie S, Hu D (2011). Timing of continuous veno-venous hemodialysis in the treatment of acute renal failure following cardiac surgery. Heart Vessel.

[48] Leite TT, Macedo E, Pereira SM, Bandeira SR, Pontes PH, Garcia AS, Militao FR, Sobrinho IM, Assuncao LM, Liborio AB (2013). Timing of renal replacement therapy initiation by AKIN classification system. Crit Care.

[49] Liu KD, Himmelfarb J, Paganini E, Ikizler TA, Soroko SH, Mehta RL, Chertow GM (2006). Timing of initiation of dialysis in critically ill patients with acute kidney injury. Clin J Am Soc Nephrol.

[50] Shiao CC, Ko WJ, Wu VC, Huang TM, Lai CF, Lin YF, Chao CT, Chu TS, Tsai HB, Wu PC (2012). U-curve association between timing of renal replacement therapy initiation and in-hospital mortality in postoperative acute kidney injury. PLoS One.

[51] Shum HP, Chan KC, Kwan MC, Yeung AW, Cheung EW, Yan WW (2013). Timing for initiation of continuous renal replacement therapy in patients with septic shock and acute kidney injury. Ther Apher Dial.

[52] Thakar CV, Christianson A, Almenoff P, Freyberg R, Render ML (2013). Degree of acute kidney injury before Dialysis initiation and hospital mortality in critically ill patients. Int J Nephrol.

[53] Wu VC, Ko WJ, Chang HW, Chen YS, Chen YW, Chen YM, Hu FC, Lin YH, Tsai PR, Wu KD (2007). Early renal replacement therapy in patients with postoperative acute liver failure associated with acute renal failure: effect on postoperative outcomes. J Am Coll Surg.

[54] Wu SC, Fu CY, Lin HH, Chen RJ, Hsieh CH, Wang YC, Yeh CC, Huang HC, Huang JC, Chang YJ (2012). Late initiation of continuous veno-venous hemofiltration therapy is associated with a lower survival rate in surgical critically ill patients with postoperative acute kidney injury. Am Surg.

[55] Tsai TT, Patel UD, Chang TI, Kennedy KF, Masoudi FA, Matheny ME, Kosiborod M, Amin AP, Weintraub WS, Curtis JP (2014). Validated contemporary risk model of acute kidney injury in patients undergoing percutaneous coronary interventions: insights from the National Cardiovascular Data Registry Cath-PCI registry. J Am Heart Assoc.

[56] Gurm HS, Dixon SR, Smith DE, Share D, Lalonde T, Greenbaum A, Moscucci M, Registry BMC (2011). Renal function-based contrast dosing to define safe limits of radiographic contrast media in patients undergoing percutaneous coronary interventions. J Am Coll Cardiol.

[57] Iakovou I, Dangas G, Mehran R, Lansky AJ, Ashby DT, Fahy M, Mintz GS, Kent KM, Pichard AD, Satler LF (2003). Impact of gender on the incidence and outcome of contrast-induced nephropathy after percutaneous coronary intervention. J Invasive Cardiol.

[58] Hsu RK, McCulloch CE, Heung M, Saran R, Shahinian VB, Pavkov ME, Burrows NR, Powe NR, Hsu CY, Centers for Disease C (2016). Exploring potential reasons for the temporal trend in Dialysis-requiring AKI in the United States. Clin J Am Soc Nephrol.

[59] Waikar SS, Curhan GC, Ayanian JZ, Chertow GM (2007). Race and mortality after acute renal failure. J Am Soc Nephrol.

[60] Cronin RM, VanHouten JP, Siew ED, Eden SK, Fihn SD, Nielson CD, Peterson JF, Baker CR, Ikizler TA, Speroff T (2015). National Veterans Health Administration inpatient risk stratification models for hospital-acquired acute kidney injury. J Am Med Inform Assoc.

[61] Waikar SS, Wald R, Chertow GM, Curhan GC, Winkelmayer WC, Liangos O, Sosa MA, Jaber BL (2006). Validity of international classification of diseases, ninth revision, clinical modification codes for acute renal failure. J Am Soc Nephrol.

[62] Grams ME, Waikar SS, MacMahon B, Whelton S, Ballew SH, Coresh J (2014). Performance and limitations of administrative data in the identification of AKI. Clin J Am Soc Nephrol.

[63] Quinn RR, Laupacis A, Austin PC, Hux JE, Garg AX, Hemmelgarn BR, Oliver MJ (2010). Using administrative datasets to study outcomes in dialysis patients: a validation study. Med Care.

[64] Romano PS, Mark DH (1994). Bias in the coding of hospital discharge data and its implications for quality assessment. Med Care.

[65] Vlasschaert ME, Bejaimal SA, Hackam DG, Quinn R, Cuerden MS, Oliver MJ, Iansavichus A, Sultan N, Mills A, Garg AX (2011). Validity of administrative database coding for kidney disease: a systematic review. Am J Kidney Dis.

